# Human-Assisted Spread of a Maladaptive Behavior in a Critically Endangered Bird

**DOI:** 10.1371/journal.pone.0079066

**Published:** 2013-12-09

**Authors:** Melanie Massaro, Raazesh Sainudiin, Don Merton, James V. Briskie, Anthony M. Poole, Marie L. Hale

**Affiliations:** 1 School of Environmental Sciences, Charles Sturt University, Albury, New South Wales, Australia; 2 Department of Mathematics and Statistics, University of Canterbury, Christchurch, New Zealand; 3 National Office, Department of Conservation, Wellington, New Zealand; 4 School of Biological Sciences, University of Canterbury, Christchurch, New Zealand; Fred Hutchinson Cancer Research Center, United States of America

## Abstract

Conservation management often focuses on counteracting the adverse effects of human activities on threatened populations. However, conservation measures may unintentionally relax selection by allowing the ‘survival of the not-so-fit’, increasing the risk of fixation of maladaptive traits. Here, we report such a case in the critically-endangered Chatham Island black robin (*Petroica traversi*) which, in 1980, was reduced to a single breeding pair. Following this bottleneck, some females were observed to lay eggs on the rims of their nests. Rim eggs left in place always failed to hatch. To expedite population recovery, rim eggs were repositioned inside nests, yielding viable hatchlings. Repositioning resulted in rapid growth of the black robin population, but by 1989 over 50% of all females were laying rim eggs. We used an exceptional, species-wide pedigree to consider both recessive and dominant models of inheritance over all plausible founder genotype combinations at a biallelic and possibly sex-linked locus. The pattern of rim laying is best fitted as an autosomal dominant Mendelian trait. Using a phenotype permutation test we could also reject the null hypothesis of non-heritability for this trait in favour of our best-fitting model of heritability. Data collected after intervention ceased shows that the frequency of rim laying has strongly declined, and that this trait is maladaptive. This episode yields an important lesson for conservation biology: fixation of maladaptive traits could render small threatened populations completely dependent on humans for reproduction, irreversibly compromising the long term viability of populations humanity seeks to conserve.

## Introduction

Humans impose considerable selection pressures on wildlife populations worldwide [1]–[3]. Environmental changes caused by humans have been shown to result in accelerated evolutionary changes of life history traits in a number of animal species [4]–[7]. For example, selective harvesting of large and old individuals through trophy hunting and fishing has resulted in populations of individuals that show reduced growth and earlier sexual maturation [4]–[6]. Human activity has been also attributed to a reduction in beak size diversity among medium ground finches (*Geospiza fortis*) at Academy Bay in the Galápagos Islands [8]. In such instances, human-induced environmental changes impose selection pressures distinct from historical and natural sources of selection [9]; these may increase a population's vulnerability to environmental stochastic events, increasing the risk of extinction [10], [11].

**Figure 1:**
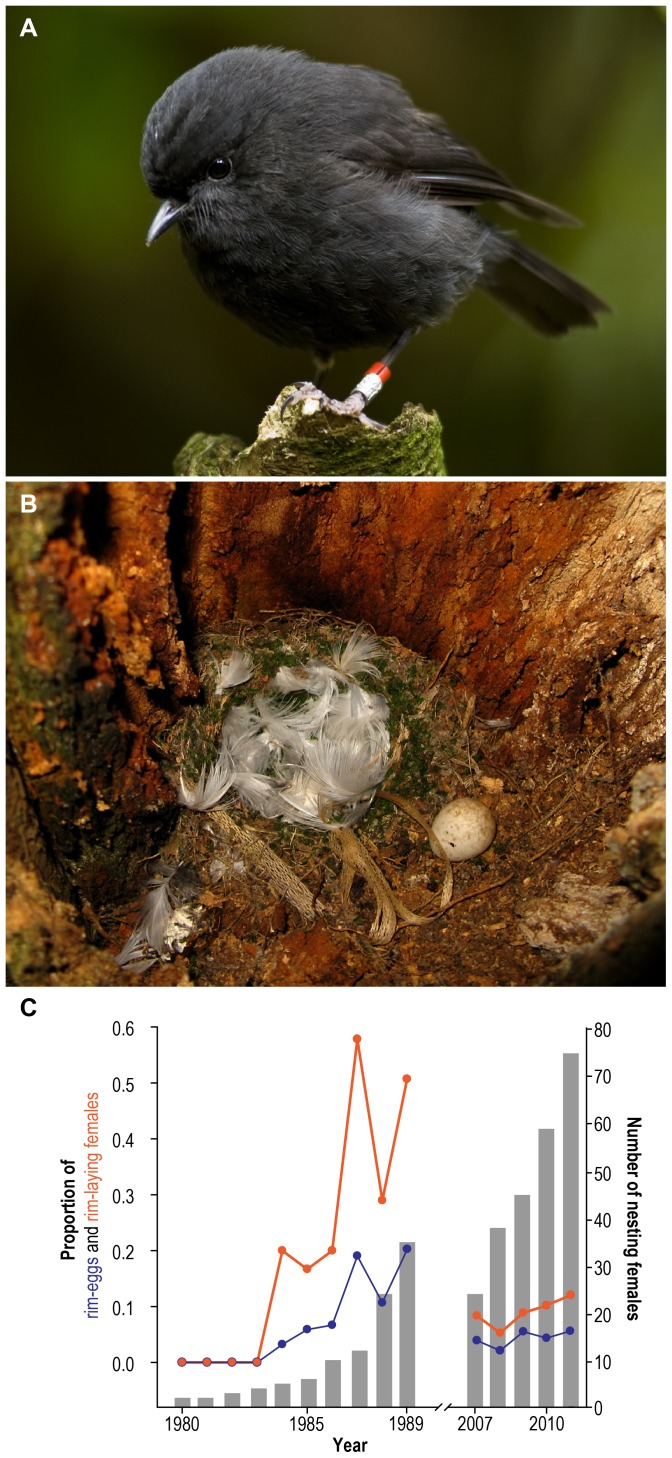
An adult black robin (*Petroica traversi*) (A); Black robin nest containing a properly placed egg inside the nest (among white petrel feathers) and a rim egg outside the nest cup (B); Proportion of rim eggs (blue line) and rim-laying females (red line) per year (C). Grey bars represent total nesting females from 1980–89 and observed nesting females from 2007–11.

In contrast, conservation measures aim to aid the recovery of endangered species. In general, conservation efforts undertaken to save the most endangered species from immediate extinction, focus, by necessity, on increasing productivity and reducing mortality. However such efforts could inadvertently alter or relax selection, potentially allowing the ‘survival of the not-so-fit’. This could in theory lead to irreversible evolutionary changes to the long term detriment of a population [12]. This is a particular concern for small populations, where deleterious mutations may become fixed due to the pervasive impact of genetic drift [13]. While the effects of deleterious mutation accumulation are evident from captive breeding programs [13]–[15], empirical studies demonstrating such a risk in wild populations are lacking. In light of the increasing number of endangered species under management around the world, developing an understanding of the long-term impacts of conservation activities on the genetic health of populations is important to prevent future extinction. Here we document the spread of a maladaptive genetic trait in the black robin (*Petroica traversi*), a critically endangered passerine (Figure 1A) endemic to the Chatham Islands, an archipelago ∼800 km east of New Zealand.

The introduction of exotic mammalian predators to the Chatham Islands was the primary cause of the extinction of several endemic birds on these islands [16], [17]. The black robin was thought to be among these extinctions until the re-discovery in 1938 of a small, isolated population of 20–35 birds on Little Mangere (Tapunaenuku) Island, an approximately 15 ha sized island which is part of the Chatham Island archipelago [18]. Following its isolation for over a century, the black robin population further declined to five individuals in 1980, including only a single breeding pair [19]. Conservation measures rescued the species from the brink of extinction, and by 1998 the population recovered to ∼200 birds. During the initial period of intense management, some females were observed to lay part or their entire clutch on the rim rather than inside their nest (Figure 1B). These ‘rim eggs’ were not incubated and without human intervention, always failed to hatch. In the 1980s, when the black robin faced imminent extinction, New Zealand Wildlife Service staff repositioned rim eggs inside nests where they could be incubated. Rim eggs were viable if incubated and the resulting increased productivity contributed to a rapid recovery of the species [19]. The aim of this study is to test the heritability of rim laying and investigate the effect of the management strategy of egg repositioning on the frequency of rim laying. We used a species-wide pedigree to show that the observed pattern of rim laying follows a model of inheritance. We also find that rim laying behavior incurs fitness costs which led to a decrease in its frequency following cessation of egg repositioning.

## Results

During the period of intensive conservation management from 1980–89, egg-laying behavior was monitored in a total of 56 females and 236 clutches. The single surviving fertile female in 1980 exhibited normal egg-laying behavior, but in 1984, one of the five females present in the population laid a single rim egg. By 1989, 51.4% of the population (18 out of 35 females) was observed to lay rim eggs (a total of 30 rim eggs, which constituted 20.4% of all eggs laid that year) (Figure 1C). During this period (1980–89), the incidence of rim laying significantly increased in the population (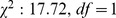, p-value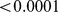).

If rim-laying has a genetic basis, and its spread has been facilitated by human intervention through egg repositioning, the frequency of this trait would be predicted to decrease following cessation of intervention. Repositioning of rim eggs ceased in 1990; we therefore compared egg-laying data from three years before cessation of repositioning (1987–89) with a three year period almost two decades after management stopped (2007–09). Consistent with relaxed selection during repositioning of rim eggs, the occurrence of rim eggs has decreased significantly between these two periods (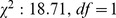, p-value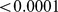) (Figure 1C). To ensure our analyses were not confounded by an island effect (data from the 1980s are from two islands, but only from Rangatira Island for the later period), we repeated all analyses including only data derived from Rangatira Island for both time periods. Exclusion of data from Mangere Island does not affect results. All analyses therefore include 1980s data from both island populations, and include the entire breeding population from 1980–89.

We next assessed whether rim-laying behavior has an observable fitness effect, using black robin breeding data from 2007–11. When rim eggs were not repositioned, females that laid rim eggs had significantly reduced clutch sizes (i.e. number of eggs laid inside nests that were incubated), and decreased hatching and breeding success compared to normal-laying females, demonstrating that rim laying substantially reduces fitness (Table 1).

**Table 1:** Fitness consequences of rim laying behavior.

Fitness	Normal-laying	Rim-laying	Statistics			
Measure	Females ( 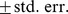 )	Females ( 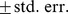 )	*n*	*χ* ^2^	df	p-value
Clutch size	2.02 (±0.03)	1.12 (±0.11)	281	10.98	1	0.0009
Hatching success	1.48 (±0.05)	0.61 (±0.12)	260	29.48	1	<0.001
Fledgling success	1.03 (±0.06)	0.50 (±0.13)	242	9.41	1	0.0021

Data from 2007–11 (during which rim eggs were not repositioned) shows that females that laid rim eggs had a significantly reduced clutch size (i.e. number of eggs laid inside nests that were incubated), and decreased hatching and breeding success compared to normal-laying females. We obtain p-values from likelihood ratio tests with generalized linear mixed models of data with sample size *n*.

The human-assisted spread (between 1984–89) and subsequent decline of rim laying following cessation of intervention (2007–11) are consistent with, but do not demonstrate a genetic basis for this trait.

**Figure 2:**
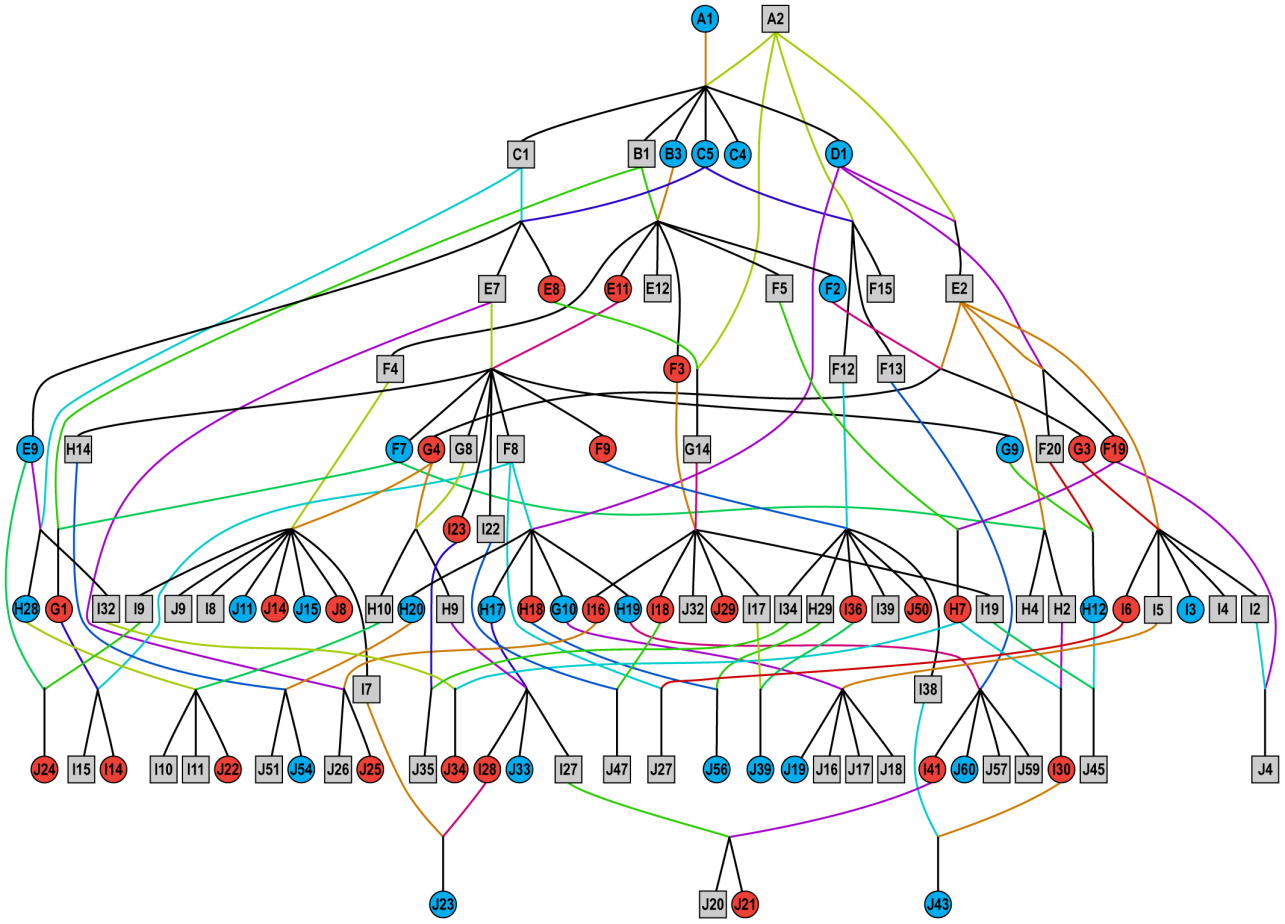
Pedigree of all black robins breeding between 1980 and 1989. All individuals are labelled and descend from one breeding pair (A1 and A2). Males are shown as squares. Females that lay rim eggs are shown as red ircles and females that do not lay rim eggs are shown as blue circles (pedigree was generated using Pedigraph [31]).

**Figure 3:**
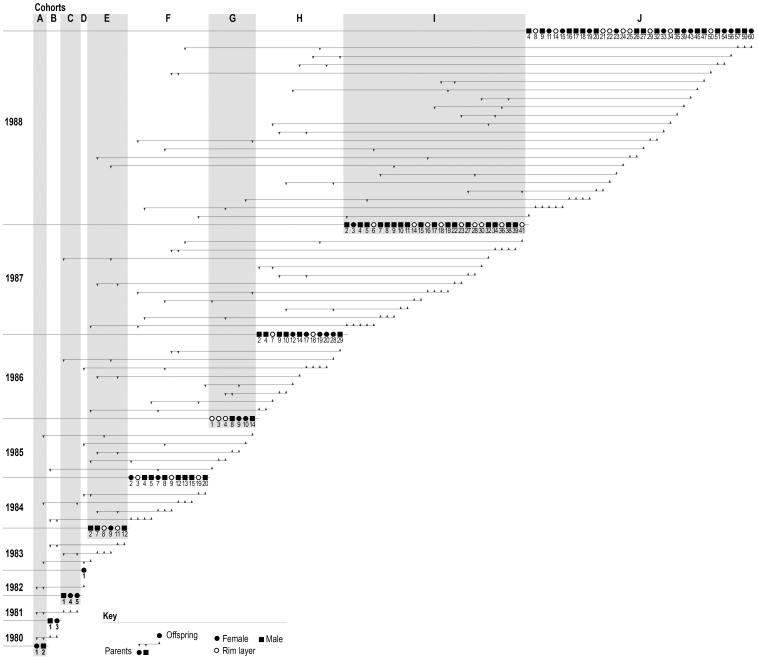
Temporally-explicit pedigree of all black robins breeding between 1980 and 1989. All individuals descend from one breeding pair (A1 and A2). Circles (females), squares (males). Offspring that successfully fledged within one year are presented as one cohort. Females that lay rim eggs are shown as white circles, females that do not lay rim eggs are presented as black circles.

To examine whether rim laying is consistent with a genetic explanation, we evaluated pedigree data from the entire black robin population from 1980–89 (Figure 2). A temporally explicit pedigree (Figure 3) illustrates the emergence and spread of this trait within the population from 1980 to 1989. The trait was not present in the founding female (A1), or her four daughters (B3, C4, C5 and D1), but appears in 15 of 24 granddaughters of the founding pair. Moreover, the high levels of inbreeding that occurred are apparent (Fig. 3): for example, the founding male (A2) not only bred successfully with the founding female (A1), but also with two of his daughters (D1, and C5) and one of this granddaughters (E8). For a full-likelihood analysis, we assumed a single locus with two alleles (A, a). We modelled the sex-limited rim laying phenotype under each of the following two possibilities: (i) dominant, where rim-laying results from expression of (A); (ii) recessive, where rim-laying results from expression of (a). The locus could be on an autosome or a sex chromosome. In birds, females are the heterogametic sex (ZW) and males are the homogametic sex (ZZ) [20]. The rim laying trait could not be located on the W chromosome as rim-laying females did not always produce rim-laying daughters, but placement on the Z chromosome is consistent with our pedigree data. To model the locus on an autosome as well as on the Z chromosome we considered four possible (parentally) ordered genotypes (aa, aA, Aa, AA). Thus for each one of the 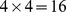 genotype combinations for the founding female (A1) and founding male (A2) we first allowed for the trait to be recessive or dominant and further allowed the locus to be on an autosome or the Z chromosome. We computed the likelihood of the observed phenotypes under each one of these 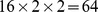 models by integrating over unobserved genotypic configurations that are consistent with the model of inheritance over all individuals in the observed pedigree using a pedigree-specific adaptation of the generic sequential Monte Carlo (SMC) algorithm [21]. Model 4 was the best on the basis of its Akaike information criterion (AIC) score given by 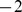 times log likelihood (Table 2) since all the models have the same number of parameters. The relative likelihood of the next best model is only about 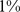. We also conducted Bayesian model selection from their posterior probabilities under the assumption that all these models have a uniform prior probability of 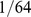. Only five of the sixty four models had a positive likelihood and their posterior probabilities are given in Table 2. The simple autosomal dominant model with founder genotypes aa X aA (or aa X Aa) has over 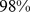 of the posterior probability. Thus, a full likelihood based model selection using AIC as well as Bayesian posterior probabilities indicates that the pattern of occurrence of rim-laying upon the pedigree is best explained by a model of simple dominant Mendelian inheritance at an autosomal biallelic locus with the male founder being heterozygous (aA) and the female founder being homozygous (aa) for the non-rim allele (Model 4 in Table 2).

**Table 2:** Model Selection.

Model	Founder Genotype	Mendelian	Chromosomal	−2× log	Posterior
Number	Female×Male	Inheritance	Location	likelihood	Probability
1	Aa×AA	Recessive	Autosome	92.250	0.0013
2	AA×Aa	Recessive	Autosome	92.428	0.0012
3	Aa×Aa	Recessive	Autosome	87.486	0.0142
4	aa×Aa	Dominant	Autosome	79.006	0.9832
5	aa×Aa	Dominant	Z	96.988	0.0001

Log likelihood and posterior probability of the phenotypes conditional on the pedigree, founder genotypes and model of inheritance. Z is a sex chromosome. The prior probability of each model is uniformly distributed and all other models considered (see text) have zero likelihood.

**Figure 4:**
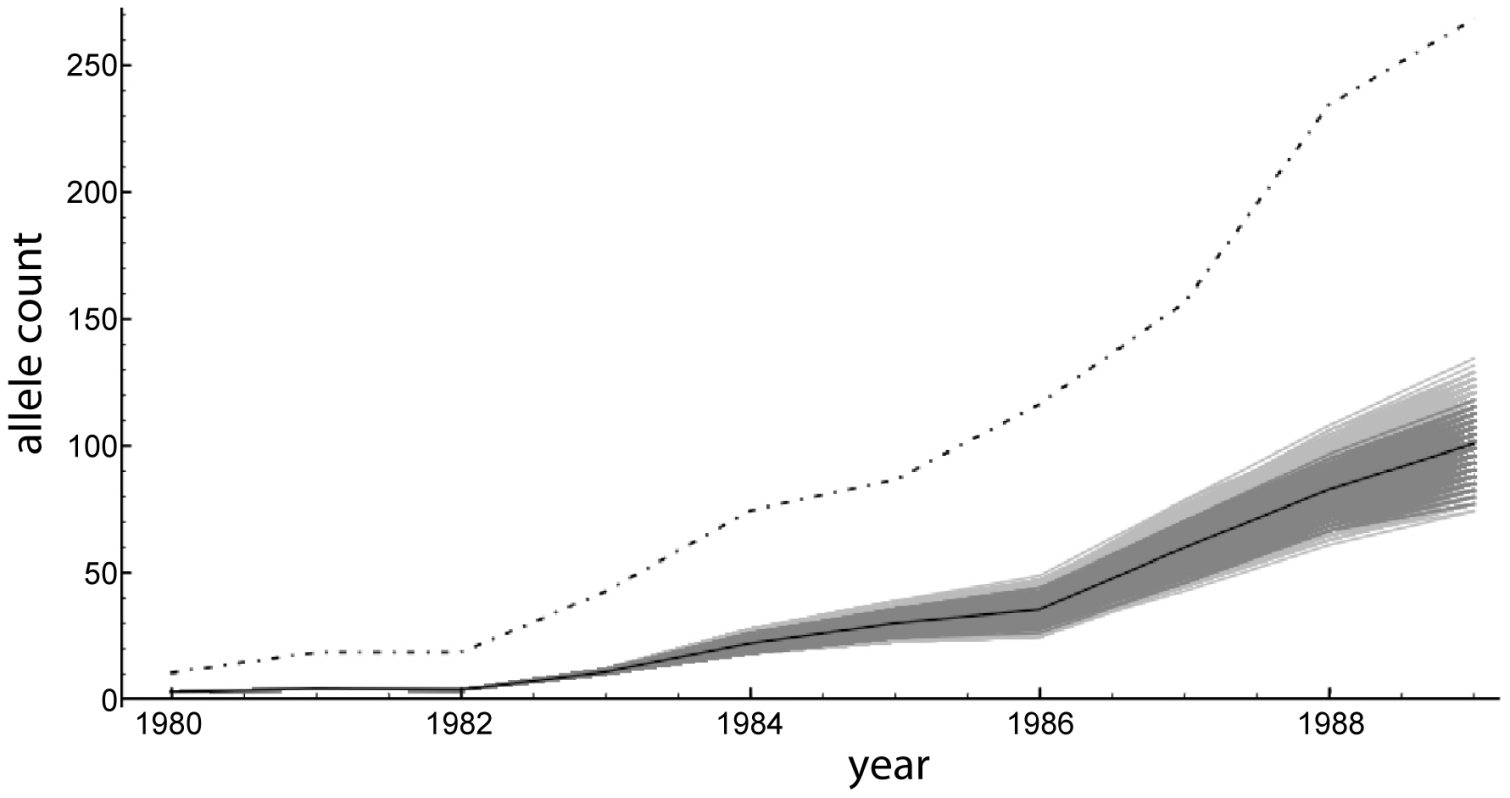
Inferred trajectories of allele A under the simple dominant model (model No. 4 in Table 2) between 1980 and 1989. The mean trajectory is shown by black line and the 50% and 95% confidence sets of the trajectories are shown in decreasing shades of grey. Twice the population size (total number of alleles) is shown by the dashed and dotted line.

To gain insights into the yearly increase of the dominant allele A under Model 4 during the managed phase between 1980 and 1989, we inferred the trajectories of the number of A alleles from the compatible genotypic configurations that are readily available from our SMC algorithm over the entire population pedigree. The mean and confidence sets of the trajectories of allele A as well as the total number of alleles (twice the population size) are given in Fig. 4. In the absence of natural selection and the presence of strong genetic drift, the expected number of A alleles increased from 1 out of 4 (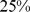) at the beginning of 1980 to 100 out of 268 (∼37%) at the end of 1989 when about half of all nesting females laid rim eggs. The 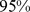 confidence set includes trajectories of allele A that are well over 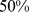 of the population by 1989.

We next sought to rule out the possibility that rim-laying is an individual response to environmental conditions. Traditional segregation analyses [22] are ideally suited for samples of pedigrees from a large population that is expected to be at some allelic equilibrium. Such methods are inappropriate to assess the genetic basis of rim laying in black robins due to the extremely strong genetic drift and complex inbreeding loops in the pedigree induced by a severe bottleneck. We therefore developed an exact phenotype permutation test that allowed us to test the null hypothesis of a non-genetic basis for rim laying using the full likelihood, from our SMC algorithm, as the test statistic. We reject the null hypothesis in favour of an alternative hypothesis based on the genetic mechanism specified by Model 4 (p-value = 0.037).

## Discussion

We have documented the emergence, and human-assisted spread of a maladaptive egg-laying behavior with a genetic basis in the endangered Chatham Island black robin. Human intervention, by relaxing selection against rim-laying individuals, facilitated a rapid increase in the frequency of this deleterious dominant trait. We surmise that the spread was facilitated by the egg repositioning that rendered this otherwise maladaptive trait effectively neutral, enabling its spread via strong genetic drift and high levels of inbreeding within an extremely small population. The human-assisted spread of this maladaptive trait highlights a dilemma that conservation efforts face worldwide: rapid population increase is crucial to avert extinction of severely threatened species, but management practices that reduce mortality and increase fecundity may inadvertently relax selection, which may in turn lead to the fixation of deleterious alleles.

Inadvertent fixation of deleterious alleles risks undermining the ultimate goal of conservation: the reestablishment of viable, self-sustaining populations in the wild. Hence, conservation planning has to overcome this fundamental dilemma of rapidly increasing the size of severely endangered populations to avoid immediate extinction but without simultaneously increasing the frequency of detrimental alleles that are already present in the population and risking their potential fixation.

Relaxation of selection following from human activity is a lesson well known from domestication, where artificial selection has made some domesticated species entirely dependent on humans for successful reproduction [23]. The total dependency of one species on another for reproduction is however an extreme outcome. The domesticated silkworm (*Bombyx mori*) provides the most stunning example of this. Over the course of 5,000 years it has become utterly dependent on humans for reproduction and survival [24]. The strange fate of the domesticated silkworm is most eloquently summarised by early Japanologist Lafcadio Hearn, who, on seeing silkworm farms for himself, wrote of the silkworm moths reserved for breeding, “They have beautiful wings, but cannot use them. They have mouths, but do not eat. They only pair, lay 10 eggs, and die. For thousands of years their race has been so well-cared for, that it can no longer take any care of itself.”[25].

In contrast, our results show that the black robin just narrowly escaped such a fate. During the initial management period, when eggs were repositioned, the rim-laying trait rose in frequency to over 50% of females. Conditions favoring relaxed selection on the rim-laying trait have, to our knowledge, been absent for two decades, and selection against rim-laying has significantly decreased the frequency of this trait (Figure 1C). However, the allele was not lost during this period, and an average of 9% of females still laid rim eggs in the period 2007–11. Alleles that are lethal or highly deleterious should be rapidly purged from small populations, even under conditions favoring drift. However, mildly deleterious alleles are less likely to be purged and far more likely to become fixed [13], [15], [26], [27]. Rim-laying, despite being a dominant trait under our best-fitted model, is only expressed in females, so the rim-laying allele is hidden from selection in males. The rim-laying allele thus persists at high frequency in the black robin population, highlighting the lasting genetic impact of human intervention, even in the face of selection against this trait. In this respect, genetic dependency of the endangered population on continued management is a genuine risk of which conservation biologists must be cognizant.

## Materials and Methods

All research was carried out with appropriate permits from the Animal Ethics Committee at the University of Canterbury and the Department of Conservation in New Zealand.

### Data collection

Chatham Island black robin populations were monitored between 1980–89 on Mangere and Rangatira Islands (two small islands part of the Chatham Island archipelago), and between 2007–11 on Rangatira Island. Birds were color-banded to allow individual recognition. Nesting attempts were monitored, recording clutch size, rim eggs and parentage of offspring. From 1980 to 1989, eggs laid on the rim were repositioned inside nests or relocated to Chatham Island tomtit (*Petroica macrocephala chathamensis*) nests to ensure that they were incubated. From 2007–11, nests were monitored to record the occurrence of rim eggs, but rim eggs were not repositioned.

### Analysis of rim-laying occurrence

Variation in occurrence of rim eggs over time was analysed using generalised linear mixed models (GLMMs) fit by the Laplace approximation (lme4 package in R v2.12.2) [28], [29]. The response variable for both GLMMs was a binomial ratio of the number of rim eggs in relation to the number of eggs laid normally per nesting attempt and we used a logit link function for model fitting. To test whether the occurrence of rim eggs varied from 1980–1989, ‘year’ was the fixed variable, while ‘period’ was the fixed variable in the GLMM testing whether the occurrence of rim eggs declined following cessation of repositioning (comparing 1987–89 with 2007–09 data). For both analyses, multiple clutches laid by the same female were accounted for by including female as a random effect. To obtain a more conservative measure of the effect of a predictor variable on the response variable, we used likelihood ratio tests (LRTs) comparing log likelihood of the GLMMs including both the fixed and the random variable ‘female’ to a simpler model including only ‘female’ as random variable.

To test whether rim laying leads to a substantial fitness loss in black robins, we again used GLMMs as described above with binomial or Poisson errors to compare effective clutch size, hatching success and breeding success of normally-laid clutches and those that included rim eggs. Female identity was included as a random effect in all analyses and LRTs were performed to obtain a more conservative measure of the effect of a predictor variable on the response variable. Parameter estimates (±1 se) of all five GLMMs are given in Table S1.

### Genetic basis of rim-laying

We use a sequential Monte Carlo (SMC) [21] algorithm over a nested increasing sequence of sub-pedigrees of the black robin population pedigree from 1980 to 1989 to compute the likelihood under each model. Our algorithm evolves a particle system of two million compatible joint genotypic configurations and thereby readily allows for insights into the hidden genotypic distribution over the entire population pedigree through time (Figure 3). The sub-pedigree sequence is constructed by a breadth-first expansion from the founding male and female. At each step in the algorithm we maintain a population of particles that specify the joint genotypic configurations over individuals in the sub-pedigree that are compatible with the observed phenotypes under a genetic model in Table 2. As the sub-pedigree expands in each iteration random genotypes for the new individuals are proposed for each particle according to the genetic model and the genotypes of its parents. Finally, the proportion of particles with joint genotypes over the new sub-pedigree that remain compatible with the observed phenotypes is used to update the likelihood. The details of this likelihood computation are given in the supplementary material (Appendix S1).

Based on this likelihood as a test statistic we develop an exact phenotype permutation test for non-genetic versus genetic factors. The phenotype permutation test is described in detail in Appendix S1 and only briefly here. Under the null hypothesis of independence between the phenotype and genotype, where each mature female has the rim laying phenotype according to an independent and identical Bernoulli random variable, the minimal sufficient statistic is given by the proportion of the rim laying females among the mature females. Thus, under the null hypothesis every permutation of the phenotypes among the mature females in the pedigree is equally likely as it will have the same sufficient statistic. We obtained a large number of random permutations of the phenotypes among the mature females in the pedigree. We compared the test statistic given by the full likelihood under Model 4 for each such permutation with that for the observed phenotypes. This test statistic is a natural measure of deviation from the null hypothesis towards the alternative hypothesis based on Model 4 as it will get larger if the alternative hypothesis becomes more likely. We estimated the p-value from the proportion of the random phenotype permutations with likelihood as high or higher than that of the observed phenotypes under Model 4. All computations were implemented using the graph libraries in Sage [30].

## Supporting Information

Table S1 **Parameter estimates (±1 se), z statistics and** ***P*** **-values of generalized linear mixed models testing whether occurrence of rim eggs varied over time (A, B) and whether rim laying incurs fitness costs (C–E).** In all models female identity was included as a random effect. *P*-values presented here are not the same than those presented in the text. In the text, *P*-values of the more conservative likelihood ratio tests are presented.
(DOC)

Appendix S1 **Pedigrees, likelihoods and tests.**
(PDF)
