# Supplementary material for: Human-Assisted Spread of a Maladaptive Behavior in a Critically Endangered Bird
Source: PLoS One. 2013 Dec 9;8(12):e79066. doi: 10.1371/journal.pone.0079066 (PMC3857173; doi:10.1371/journal.pone.0079066)
Supplement: Table S1 — Parameter estimates (±1 se), z statistics and P -values of generalized linear mixed models testing whether occurrence of rim eggs varied over time (A, B) and whether rim laying incurs fitness costs (C–E). In all models female identity was included as a random effect. P-values presented here are not the same than those presented in the text. In the text, P-values of the more conservative likelihood ratio tests are presented. (DOC) [file pone.0079066.s001.doc]

**Table S1.** Parameter estimates (± 1 se), z statistics and *P*-values of generalized linear mixed models testing whether occurrence of rim eggs varied over time (A, B) and whether rim laying incurs fitness costs (C-E). In all models female identity was included as a random effect. *P*-values presented here are not the same than those presented in the text. In the text, *P*-values of the more conservative likelihood ratio tests are presented.

**A.** The incidence of rim-laying increased significantly over time (1980-89).

| Fixed effects | Estimate | Std. Error | z value | *P* |
| --- | --- | --- | --- | --- |
| Intercept | -832.2988 | 227.6822 | -3.656 | 0.00026 |
| year | 0.4177 | 0.1145 | 3.647 | 0.00027 |

**B.** The frequency of rim-laying declined following cessation of repositioning. (comparing 1987-89 with 2007-10 data).

| Fixed effects | Estimate | Std. Error | z value | *P* |
| --- | --- | --- | --- | --- |
| Intercept | -1.7789 | 0.1870 | -9.513 | < 0.0001 |
| period | -1.5946 | 0.4026 | -3.961 | < 0.0001 |

**C.** Clutch size (i.e. the number of eggs laid inside nests that were incubated) varied between rim-laying females and those that lay normally.

| Fixed effects | Estimate | Std. Error | z value | *P* |
| --- | --- | --- | --- | --- |
| Intercept | 0.7009 | 0.0440 | 15.922 | < 0.0001 |
| Rim-laying females | -0.5876 | 0.1940 | -3.028 | 0.0025 |

**D.** Hatching success varied between clutches of rim-laying females and those of normal laying females.

| Fixed effects | Estimate | Std. Error | z value | *P* |
| --- | --- | --- | --- | --- |
| Intercept | 1.1227 | 0.1345 | 8.347 | < 0.0001 |
| Rim-laying females | -2.0128 | 0.3805 | -5.290 | < 0.0001 |

**E.** Breeding success varied between rim-laying females and normal laying females.

| Fixed effects | Estimate | Std. Error | z value | *P* |
| --- | --- | --- | --- | --- |
| Intercept | 0.0780 | 0.1474 | 0.530 | 0.5964 |
| Rim-laying females | -1.2768 | 0.4248 | -3.006 | 0.0027 |
