# Supplementary material for: Human-Assisted Spread of a Maladaptive Behavior in a Critically Endangered Bird
Source: PLoS One. 2013 Dec 9;8(12):e79066. doi: 10.1371/journal.pone.0079066 (PMC3857173; doi:10.1371/journal.pone.0079066)
Supplement: Appendix S1 — Pedigrees, likelihoods and tests. (PDF) [file pone.0079066.s002.pdf]

Supporting Information for 'Human-assisted spread of a maladaptive behavior in a critically endangered bird'

Melanie Massaro[1 ⋆ ∗], Raazesh Sainudiin[2 ⋆], Don Merton[3], James V. Briskie[4], Anthony M. Poole[4], Marie L. Hale[4]

1 Melanie Massaro School of Environmental Sciences, Charles Sturt University, Albury, New South Wales, Australia

2 Department of Mathematics and Statistics, University of Canterbury, Christchurch, New Zealand

3 Deceased

4 School of Biological Sciences, University of Canterbury, Christchurch, New Zealand

⋆ Melanie Massaro and Raazesh Sainudiin share first authorship.

∗ To whom correspondence should be addressed. Email: mmassaro@csu.edu.au

Appendix S1: Pedigrees, likelihoods and tests

We first introduce the notation needed to define our pedigree, genotypes, phenotypes and models of Mendelian inheritance. Then we introduce the sequential Mote Carlo method to compute the likelihood of the phenotypes under a model of inheritance over a given pedigree. Finally we explain the phenotype permutation test.

Pedigree

A pedigree is defined here to be a mathematical graph over a set of vertices $\mathbb{V}$ and a set of directed edges $\mathbb{E}$ that is given by a subset of ordered pairs in $\mathbb{V}^2 := \mathbb{V} \times \mathbb{V}$. Here $\mathbb{V}$ is the set of individuals in the population and a directed edge $(u, v) \in \mathbb{E}$ exists if and only if u is one of the parents of v and $(u, v) \in \mathbb{V}^2$. We partition $\mathbb{V}$ into $\mathbb{M}$, $\mathbb{F}$ and $\mathbb{U}$, denoting males, females and individuals of unknown sex, respectively. Let $\mathcal{G} := (\mathbb{V}, \mathbb{E})$ be the observed pedigree of the entire population during the managed phase (1980-89). Let the female and male founders be $\mathbb{A} := \{\mathsf{A1}, \mathsf{A2}\}$. For an individual $u \in \mathbb{V} \setminus \mathbb{A}$ let M_u and F_u denote its mother and father, respectively.

Phenotypes

Let $\mathbb{L}$ be the set of mature females who were observed to lay eggs and let $|\mathbb{L}|$ denote its size. Thus, $\mathbb{L}$ is a subset of $\mathbb{F}$. Let $\phi : \mathbb{L} \to \{0, 1\}$ denote a particular mapping of the females in $\mathbb{L}$ to the Bernoulli set of two behavioral phenotypes:

$$\text{for every } u \in \mathbb{L}, \quad \phi(u) = \begin{cases} 1 & \text{if } u \text{ lays rim eggs} \\ 0 & \text{if } u \text{ lays normal eggs} \end{cases} \tag{1}$$

Let the phenotypes of a set of individuals $\mathbb{U} := \{u_1, \ldots, u_m\}$ be $\phi(\mathbb{U}) := \{\phi(u) : u \in \mathbb{U}\} = \{\phi(u_1), \ldots, \phi(u_m)\}$.

Genotypes

Let $\Gamma = \{(a, a), (a, A), (A, a), (A, A)\}$ denote the set of possible ordered biallelic genotypes an individual can have. We denote the genotype of an individual u by $\gamma(u) \in \Gamma$ and its maternal and paternal copies by $\gamma_m(u)$ and $\gamma_f(u)$, respectively, i.e., $\gamma(u) = (\gamma_m(u), \gamma_f(u))$. We let Γ be a quadruple instead of the usual triple $\{(a, a), (a, A), (A, A)\}$ in order to simplify our Monte Carlo algorithm and easily encode both autosomal and sex-linked models of Mendelian inheritance. Let the genotypes of a set of individuals $\mathbb{U} := \{u_1, \ldots, u_m\}$ be $\gamma(\mathbb{U}) := \{\gamma(u) : u \in \mathbb{U}\} = \{\gamma(u_1), \ldots, \gamma(u_m)\} \in \Gamma^{\mathbb{U}}$ and that of the founders be $\gamma(\mathbb{A}) \in \Gamma^{\mathbb{A}}$. Note that $|\Gamma^{\mathbb{U}}| = 4^{|\mathbb{U}|}$.

Models of Inheritance

Let $\mathcal{M}_h^c$ denote a family of four models of Mendelian inheritance where $h \in \{D, R\}$ allows for a dominant (D) allele A or for a recessive (R) allele a and $c \in \{\alpha, z\}$ allows for the locus to be on an autosome (α) or the sex chromosome (z). We thus consider four simple models of Mendelian inheritance at a biallelic locus. The probability of an individual u's genotype $\gamma(u) = (\gamma_m(u), \gamma_f(u))$ given the model $\mathcal{M}_h^c$ and parental genotypes $\gamma(M_u) = (\gamma_m(M_u), \gamma_f(M_u))$ and $\gamma(F_u) = (\gamma_m(F_u), \gamma_f(F_u))$ is given by the independent and equal probabilities of inheriting either allele from each parent. Under the sex-linked model $\mathcal{M}_h^z$ the inheritance rules are more complex. In birds, males are ZZ and females are ZW. Thus, if u is a male, he has to inherit a copy of Z from his mother and if u is female then she has to inherit her copy of W from her mother and therefore:

$$\Pr\left(\gamma(u) \mid \gamma(M_u), \gamma(F_u), \mathcal{M}_h^c\right) = \begin{cases} \frac{1}{4} & \text{if } c = \alpha, \gamma_m(u) \in \{\gamma_m(M_u), \gamma_f(M_u)\}, \\ & \quad \gamma_f(u) \in \{\gamma_m(F_u), \gamma_f(F_u)\} \\ \frac{1}{2} & \text{if } c = z, \gamma_m(u) = \gamma_f(M_u), \\ & \quad \gamma_f(u) \in \{\gamma_m(F_u), \gamma_f(F_u)\}, u \in \mathbb{M} \\ \frac{1}{2} & \text{if } c = z, \gamma_m(u) = \gamma_m(M_u), \\ & \quad \gamma_f(u) \in \{\gamma_m(F_u), \gamma_f(F_u)\}, u \in \mathbb{F} \\ 0 & \text{otherwise.} \end{cases} \tag{2}$$

We can use (2) recursively on the pedigree starting from a given founder genotypes and propose genotypes independently for each offspring given its parental genotypes and the model of inheritance.

Finally the probability of $\phi(u)$, the phenotype for an individual u, given its genotype $\gamma(u)$ and the model of inheritance $\mathcal{M}_h^c$ is the following:

$$\Pr\left(\phi(u) \mid \gamma(u), \mathcal{M}_h^c\right) = \begin{cases} 1 & \text{if } \phi(u) = 1, \gamma(u) \in \Gamma \setminus \{(a,a)\}, h = D, u \in \mathbb{L} \\ 1 & \text{if } \phi(u) = 0, \gamma(u) \in \{(a,a)\}, h = D, u \in \mathbb{L} \\ 1 & \text{if } \phi(u) = 0, \gamma(u) \in \Gamma \setminus \{(a,a)\}, h = R, u \in \mathbb{L} \\ 1 & \text{if } \phi(u) = 1, \gamma(u) \in \{(a,a)\}, h = R, u \in \mathbb{L} \\ 1 & \text{if } u \notin \mathbb{L} \\ 0 & \text{otherwise.} \end{cases} \tag{3}$$

Since $\phi(u)$ is unobservable if u is not an egg-laying female, i.e., $u \notin \mathbb{L}$, we set $\Pr(\phi(u)) = 1$ as in [1] so that it will not affect the likelihood of the observable phenotypes.

Likelihood of Observed Phenotypes on Pedigree

We want to compute the probability of the inheritance model $\mathcal{M}_h^c$ and the founder genotypes $\gamma(\mathbb{A})$ given $\mathcal{G}$, the observed pedigree, and $\phi(\mathbb{V})$, the observed phenotypes over the nodes in $\mathcal{G}$:

$$\Pr\left(\mathcal{M}_h^c, \gamma(\mathbb{A}) \mid \mathcal{G}, \phi(\mathbb{V})\right) = \frac{\Pr\left(\phi(\mathbb{V}) \mid \mathcal{G}, \mathcal{M}_h^c, \gamma(\mathbb{A})\right) \Pr\left(\mathcal{M}_h^c, \gamma(\mathbb{A})\right)}{\sum_{\substack{c \in \{a,z\} \\ h \in \{D,R\} \\ \gamma(\mathbb{A}) \in \Gamma^{\mathbb{A}}}} \Pr\left(\phi(\mathbb{V}) \mid \mathcal{G}, \mathcal{M}_h^c, \gamma(\mathbb{A})\right) \Pr\left(\mathcal{M}_h^c, \gamma(\mathbb{A})\right)} \tag{4}$$

Note that $\Pr\left(\mathcal{G} \mid \mathcal{M}_h^c, \gamma(\mathbb{A})\right) = \Pr\left(\mathcal{G}\right)$ due to the assumed independence of the model of inheritance and the pedigree and therefore cancels out from the posterior probability of $\mathcal{M}_h^c$ and $\gamma(\mathbb{A})$ given by (4). We assume a uniform prior probability $\Pr\left(\mathcal{M}_h^c, \gamma(\mathbb{A})\right) = \frac{1}{64}$ over the $2 \times 2 \times 4^2 = 64$ possible models in the family:

$$\left\{(\mathcal{M}_h^c, \gamma(\mathbb{A})) : c \in \{a, z\}, h \in \{D, R\}, \gamma(\mathbb{A}) \in \Gamma^{\mathbb{A}}\right\} \ .$$

Next we describe how $\Pr\left(\phi(\mathbb{V}) \mid \mathcal{G}, \mathcal{M}_h^c, \gamma(\mathbb{A})\right)$, the likelihood in (4), is computed. Our algorithm is a sequential Monte Carlo [2] estimate of the likelihood given by the peeling algorithm [1] with the additional insightful feature of allowing us access to the hidden space through a Monte Carlo population of two million particles from the space of joint genotypic configurations over individuals in the pedigree such that they are compatible with the observed phenotypes under the given model $\mathcal{M}_h^c$. The sampling strategy involves sequentially growing the pedigree as a nested and increasing family of sub-graphs via a breadth-first expansion from the founders $\mathsf{A1}$ and $\mathsf{A2}$.

Let $\mathcal{G}[\mathbb{V}_t]$ denote the sub-pedigree of $\mathcal{G}$ induced by the vertices $\mathbb{V}_t$. Let $\mathbb{V}_0 = \{\mathsf{A1}, \mathsf{A2}\}$ be the vertex set of the founding male and female. Let $\mathbb{A} = \mathbb{V}_0 \subset \mathbb{V}_1 \subset \cdots \subset \mathbb{V}_T = \mathbb{V}$ denote the increasing sequence of vertices in $\mathcal{G}$ obtained by a breadth-first expansion from $\mathbb{V}_0$ that satisfies the condition that for each $u \in \bar{\mathbb{V}}_t := \mathbb{V}_t \setminus \mathbb{V}_{t-1}$ its parents M_u and F_u are in $\mathbb{V}_{t-1}$ for every $t \in \{1, 2, \ldots, T\}$. The likelihood of interest $\Pr\left(\phi(\mathbb{V}) \mid \mathcal{G}, \mathcal{M}_h^c, \gamma(\mathbb{A})\right) = \Pr\left(\phi(\mathbb{V}_T) \mid \mathcal{G}[\mathbb{V}_T], \mathcal{M}_h^c, \gamma(\mathbb{V}_0)\right)$. For each t, by the law of total probability,

$$\Pr\left(\phi(\mathbb{V}_t) \mid \mathcal{G}[\mathbb{V}_t], \mathcal{M}_h^c, \gamma(\mathbb{V}_0)\right) = \sum_{\gamma(\mathbb{V}_t) \in \Gamma^{\mathbb{V}_t}} \Pr\left(\phi(\mathbb{V}_t), \gamma(\mathbb{V}_t) \mid \mathcal{G}[\mathbb{V}_t], \mathcal{M}_h^c, \gamma(\mathbb{V}_0)\right) \ .$$

And for a given $\gamma(\mathbb{V}_t) \in \Gamma^{\mathbb{V}_t}$ we have the convenient recursive structure,

$$\Pr\left(\phi(\mathbb{V}_t), \gamma(\mathbb{V}_t) \mid \mathcal{G}[\mathbb{V}_t], \mathcal{M}_h^c, \gamma(\mathbb{V}_0)\right) = \prod_{u \in \bar{\mathbb{V}}_t} Q(u) \Pr\left(\phi(\mathbb{V}_{t-1}), \gamma(\mathbb{V}_{t-1}) \mid \mathcal{G}[\mathbb{V}_{t-1}], \mathcal{M}_h^c, \gamma(\mathbb{V}_0)\right) \quad ,$$

where,

$$Q(u) = \Pr\left(\phi(u) \mid \gamma(u), \mathcal{M}_h^c\right) \Pr\left(\gamma(u) \mid \gamma(M_u), \gamma(F_u), \mathcal{M}_h^c\right) \quad .$$

Due to this recursive likelihood decomposition over the increasing pedigree sequence $\{\mathcal{G}[\mathbb{V}_t]\}_{t=0}^{T}$, we can use the following sequential Monte Carlo algorithm to compute the likelihood of interest by a straightforward adaptation of [2] in the spirit of [3] but over a nested sequence of pedigrees.

Sequential Monte Carlo Algorithm

- At $t = 0$, initialize all particles at the founder nodes to have the given founder genotypes.
  `FOR` $i = 1, \ldots, N$; `DO`:
  - $\gamma_0^{(i)}(u) \leftarrow \gamma(u)$, for each founder $u \in \mathbb{V}_0 = \mathbb{A}$
  - $W_0^{(i)} \leftarrow \prod_{u \in \mathbb{V}_0} \Pr(\phi(u) \mid \gamma_0^{(i)}(u), \mathcal{M}_h^c)$
- `FOR` $t = 1, \ldots, T$; `DO`:
  - Resample to obtain $\left\{ \frac{1}{N}, \left(\hat{\gamma}_{t-1}^{(i)}(u) : u \in \mathbb{V}_{t-1} \right) \right\}_{i=1}^{N}$
  - `FOR` $i = 1, \ldots, N$; `DO`:
    * Extend $\left\{ \hat{\gamma}_{t-1}^{(i)}(u) : u \in \mathbb{V}_{t-1} \right\}$ to $\left\{ \gamma_t^{(i)}(u) : u \in \mathbb{V}_t \right\}$ by proposing genotypes for each new offspring node in $\mathbb{V}_t \setminus \mathbb{V}_{t-1}$ according to $\mathcal{M}_h^c$ and the genotypes of its parent nodes in $\mathbb{V}_{t-1}$ specified by the sub-pedigree $\mathcal{G}[\mathbb{V}_t]$.
    * $W_t^{(i)} \leftarrow \prod_{u \in \mathbb{V}_t \setminus \mathbb{V}_{t-1}} \Pr(\phi(u) \mid \gamma_t^{(i)}(u), \mathcal{M}_h^c)$
- We can estimate $\Pr\left(\mathcal{G}, \phi(\mathbb{V}) \mid \mathcal{M}_j^c, \gamma(\mathbb{A})\right)$, the needed probability, by $\prod_{t=0}^{T} \hat{Z}_t$, with $\hat{Z}_t = \frac{1}{N} \sum_{i=1}^{N} W_t^{(i)}$.

Phenotype Permutation Test

Our statistical test requires us to compute $P_0(\phi(\mathbb{V})) := P_0\left(\{\phi_o(u) : u \in \mathbb{V}\}\right)$, the probability of phenotypes under a null hypothesis H_0 that is independent of the pedigree and thereby devoid of any notion

of genetic inheritance. Under the null hypothesis H_0 we model the phenotypes $\phi(\mathbb{V})$ using independent and identical Bernoulli(θ) random variables with the probability of laying rim eggs given by $\theta \in (0,1)$. In other words, the probabilities under H_0 for the two phenotypes 1 and 0 are $P_0(\phi(u)=1)=\theta$ and $P_0(\phi(u)=0)=1-\theta$, respectively. Thus the observed phenotypes specified by the set $\phi_o(\mathbb{L}) := \{\phi_o(u) : u \in \mathbb{L}\}$ is modeled to be realized under H_0 with probability:

$$\begin{aligned} P_0(\phi(\mathbb{L})) &:= P_0\left(\{\phi_o(u) : u \in \mathbb{L}\}\right) \\ &= \prod_{u \in \mathbb{L}} P_0(\phi_o(u)) \\ &= \theta^{\sum_{u\in\mathbb{L}} \phi_o(u)} (1-\theta)^{|\mathbb{L}| - \sum_{u\in\mathbb{L}} \phi_o(u)} \end{aligned} \tag{5}$$

For the unobservable case, i.e., when $u \in \mathbb{V} \setminus \mathbb{L}$, we set $P_0(\phi(u)) = 1$ so that $P_0(\phi(\mathbb{V})) = P_0(\phi(\mathbb{L}))$. Therefore, under the null hypothesis H_0 the likelihood of the phenotypes $\phi_o(\mathbb{L})$ only depends on the size of $\mathbb{L}$ and the number of rim-laying females in $\mathbb{L}$, i.e., $\ell = |\mathbb{L}|$ and $r = \sum_{u\in\mathbb{L}} \phi_o(u)$ are the sufficient statistics under H_0. Note that H_0 is fundamentally a hypothesis of non-heritability (what we mean by "environmental" or "non-genetic" here) as it completely ignores the underlying pedigree and thereby any basis for heritability of the rim-laying phenotype. Under H_0 every phenotype map $\phi(u) : \mathbb{L} \to \{0,1\}$ in $\{0,1\}^{\mathbb{L}}$ such that,

$$\left(|\mathbb{L}|, \sum_{u\in\mathbb{L}} \phi(u)\right) = \left(|\mathbb{L}|, \sum_{u\in\mathbb{L}} \phi_o(u)\right) = (\ell, r) \ , \tag{6}$$

is equally likely. The set of such phenotypes can be constructed by first taking each permutation of the vertex labels in $\mathbb{L}$ and then assigning phenotye 1 to the first r (and phenotype 0 to the last $\ell - r$) vertex labels of this permutation. The basic idea behind our phenotype permutation test is identical to that behind classical permutation test of the null hypothesis: two samples are drawn from the same distribution, except that we need meaningful test statistics that can take specific notions of inheritance on the observed pedigree (that relates vertices or individuals in $\mathbb{L}$ under an alternative hypothesis) into account. In particular, we would like the test statistic $T = T(\phi)$ to take larger values if the alternative hypothesis H_a that specifies a law of inheritance on the observed pedigree becomes more likely. Then

the P-value of rejecting H_0 in favor of a given alternative hypothesis of inheritance H_a is given by

$$\begin{aligned} P_0(T(\phi) \geq T(\phi_o)) &= P_0(\{\phi : T(\phi) \geq T(\phi_o)\}) \\ &= \frac{1}{\ell!} |\{\phi : T(\phi) \geq T(\phi_o)\}| \ . \end{aligned}$$

We take T to be the likelihood under our Model 4, the model of inheritance with the highest posterior probability. This likelihood will become large when the alternate hypothesis of Model 4 is true and we can obtain its null distribution from the permuted phenotypes that are equally likely when the null hypothesis is true. Note that we cannot do a likelihood ratio test between the null model of independent Bernoulli trials and the alternate Model 4 since they are not nested. Finally we use a Monte Carlo estimate of the P-value from the proportion of test statistics that are greater than or equal to the observed test statistic in a random sample of 10000 phenotype permutations that are equally likely under H_0.

References

1. Cannings C, Thompson EA, Skolnick MH (1978) Probability functions on complex pedigrees. Advances in Applied Probability 10: 26-61.

2. Doucet A, Johansen AM (2009) A Tutorial on Particle Filtering and Smoothing: Fifteen Years Later. In: Crisan D, Rozovsky B, editors, The Oxford Handbook of Nonlinear Filtering, Oxford University Press.

3. Johansen AM, Del Moral P, Doucet A (2006) Sequential monte carlo samplers for rare events. In: Proceedings of the 6th International Workshop on Rare Event Simulation. pp. 256-267.
